# Downregulation of Lung Toll-Like Receptor 4 Could Effectively Attenuate Liver Transplantation-Induced Pulmonary Damage at the Early Stage of Reperfusion

**DOI:** 10.1155/2015/383907

**Published:** 2015-09-29

**Authors:** Xinjin Chi, Weifeng Yao, Ailan Zhang, Mian Ge, Jun Cai, Shaoli Zhou, Zhengyuan Xia, Gangjian Luo, Ziqing Hei

**Affiliations:** ^1^Department of Anesthesiology, Third Affiliated Hospital, Sun Yat-sen University, Guangzhou, Guangdong 510630, China; ^2^Department of Anesthesiology, The University of Hong Kong, Pokfulam, Hong Kong

## Abstract

Acute lung injury (ALI) is a severe complication of orthotopic liver transplantation (OLT) with unclear underline mechanism. Toll-like receptor 4 (TLR4) has been identified as a key receptor mediating inflammation. We hypothesized that TLR4-mediated pulmonary inflammation may contribute to development of ALI during OLT. Patients with or without ALI were observed for serum cytokines and expression of TLR4 on peripheral blood polymorphonuclear leukocytes (PMNs). Next, rats which underwent orthotopic autologous liver transplantation (OALT) were divided into sham and model groups. Pulmonary function and the level of TLR4 expression and cytokines were analyzed. Furthermore, the role of TLR4 in OALT-mediated ALI was assessed in rats treated with TLR4-siRNA before OALT. The PMNs TLR4 expression and the serum TNF-*α* and IL-*β* level were higher in patients with ALI than those with non-ALI. Interestingly, lung TLR4 expression was significantly increased after 8 hours of OALT with increased levels of TNF-*α* and IL-*β*, which lead to lung pathological damage and an increase of lung myeloperoxidase content. Moreover, knockdown of TLR4 reduced lung cytokines release and reversed the above pathologic changes after OALT and finally improved rats' survival rate. In conclusion, TLR4 overexpression, potentially by stimulating proinflammatory cytokine overproduction, contributes to the development of ALI after OLT.

## 1. Introduction

Liver transplantation is a major surgical procedure that is associated with a high incidence of postoperative complications [1]. Acute lung injury (ALI) is a common complication after orthotopic liver transplantation (OLT) that occurs in 34.2%–77.8% of patients receiving OLT and in 7.8%–23.6% of patients with post-OLT ALI aggravated by acute respiratory distress syndrome (ARDS), which itself carries a mortality rate of 76.5% [2–4]. Overwhelming inflammation has been shown to play an important role in the progress of ALI induced by OLT [5], but the initiative mechanism of inflammation remains unclear.

Toll-like receptor 4 (TLR4) is an important member of the TLR family and can be activated by noxious stimuli to initiate nuclear factor-*κ*B (NF-*κ*B) signaling and a downstream inflammatory response [6, 7]. Overexpression of TLR4, which is induced by different factors, including ischemia-reperfusion, endotoxin, trauma, and mechanical ventilation, has been shown to play a critical role in ALI [8–11]. TLR4 has also been shown to be involved in ischemia-reperfusion injury pathophysiology in certain important organs such as the liver and kidney as well as in the heart [12, 13]. At present, there is no evidence to support the important role of TLR4 in ALI after OLT. Therefore, a clarification of the role played by TLR4 may help to explain the mechanism of ALI induced by liver transplantation and may also contribute to the development of effective intervention measures for ALI.

The purposes of the current study are (1) to observe the neutrophil TLR4 expression in patients to provide clues for TLR4 involved in ALI after OLT; (2) to dynamically investigate the changes in lung pathology, TLR4 expression, and inflammatory mediators in lung tissue during orthotopic autologous liver transplantation (OALT) in a rat model; (3) to evaluate the relative importance of TLR4 in the development of ALI during OLT using RNA interference (RNAi), which is an established method for inhibiting target gene expression in a rat model [14, 15].

## 2. Materials and Methods

### 2.1. Recruitment of Patients Undergoing OLT and Anesthesia Procedure

This study was approved by the Research Ethics Board of the Third Affiliated Hospital, Sun Yat-sen University, China (number ChiCTR-OCH-12002255). Informed consent was obtained from every patient and their relatives. Twenty-two ASA III or IV patients of both sexes (19 males, 3 females), 33–59 years of age, weighing 49–74 kg, and undergoing OLT, were studied. The anesthesia procedures and management were performed by routine protocol at our hospital [4].

### 2.2. Diagnosis for Acute Lung Injury

All of the patients received modified piggyback liver transplantation, a liver transplantation technique of side-to-side caval anastomosis during orthotopic hepatic transplantation without inferior vena caval occlusion. Diagnosis of acute lung injury (ALI) [16] was based on clinical findings, and diagnosis criteria were as follows: acute onset, PaO_2_/FiO_2_ ⩽ 300, bilateral pulmonary infiltrates detected by X-ray, and pulmonary wedge pressure (PCWP) ⩽ 18 mmHg or no clinical evidence of congestive heart failure.

### 2.3. Blood Sample Collected from Recipients

The peripheral arterial blood samples (4 mL) were collected for measurement of TLR4 expression and concentration of TNF-*α* and IL-1*β* at T_1_ (after induction of anesthesia), T_2_ (1 hr after anhepatic phase), T_3_ (4 hrs after graft reperfusion), and T_4_ (24 hrs after graft reperfusion). 2 mL of the blood sample was stored in the EDTA anticoagulant tube for flow cytometry for detection of TLR4 expression on the surface of PMNs cells and the other 2 mL was centrifuged at 3000 r/min for 10 min and then stored in −30°C for assay of serum cytokines TNF-*α* and IL-1*β*.

### 2.4. Animals

Male Sprague-Dawley (SD) rats, aged 8–10 weeks and weighing 220–250 g, were purchased from* Medical Experimental Animal Center of Guangdong Province*. All the animal research protocols used in this study were approved by* the Institutional Animal Care and Use Committee of Sun Yat-sen University* in Guangzhou, China.

### 2.5. siRNA Preparation, Screening, and Delivering

Three 21-nt siRNA duplexes targeting rat TLR4 gene (number NM 019178) were designed using the siRNA Target Finder and Design Tool available at http://www.ambion.com and were commercially obtained from Ribobio (Guangzhou, China). Green fluorescent protein (GFP) siRNA, with no homology to TLR4 gene, was taken as siRNA control. TLR4 siRNA was found to have no toxic effect on hepatic stellate cells (HSC) using CCK-8 assay (data not showed). After transfection and incubation for 48 hrs, hepatic stellate cells (HSC) were harvested and expression of TLR4 at both mRNA and protein levels was detected by real-time PCR (RT-PCR) and Western blot, respectively. TLR4 siRNA with maximum inhibition rate was selected for* in vivo* experiments:

 TLR4 siRNA1, target sequence 1: GCATAGAGGTACTTCCTAA.
 5′-GCAUAGAGGUACUUCCUAA dTdT-3′ (sense).
 3′-dTdT CGUAUCUCCAUGAAGGAUU-5′ (antisense).
 TLR4 siRNA2, target sequence 2: GCTTATATCCTTAAAGAAA.
 5′-GCUUAUAUCCUUAAAGAAA dTdT-3′ (sense).
 3′-dTdT CGAAUAUAGGAAUUUCUUU-5′ (antisense).
 TLR4 siRNA3, target sequence 3: CCTAGAACATGTGGATCTT.
 5′-CCUAGAACAUGUGGAUCUU dTdT-3′ (sense).
 3′-dTdT GGAUCUUGUACACCUAGAA-5′ (antisense).

After anesthesia with pentobarbital sodium (30 mg/kg, i.p.), rats received intratracheal injection of 500 *μ*L of normal saline (NS group), 500 *μ*L (50 nmol) of siRNA vector or TLR4 siRNA (NC and siRNA group). 48 hrs later, animals in the NS, NC, and siRNA group received orthotopic autologous liver transplantation (OALT) while rats in sham group only received laparotomy and vessels separation without OALT.

### 2.6. Experiment Design

In first part of the experiment, twenty-two patients were divided into ALI group and non-ALI group according to their postoperative diagnosis after OLT surgery. In second part of the experiment, the rat OALT model was established according to our [17] and Jin et al.'s [18] previous study. The entire anhepatic phase lasted for 20 ± 1 min. Rats were randomly divided into two groups. Rats in Group S (sham operated, *n* = 16) received abdominal operation and liver dissection under anesthesia, without cold perfusion and reperfusion. Rats in OALT model group (Group M, *n* = 16) all received OALT using the above-described method [17, 18]. In Groups S and M, rats were randomly killed at 4, 8, 16, and 24 hrs after liver reperfusion (i.e., Groups M1, M2, M3, M4, resp.), and rats in Group S were killed at corresponding times after liver liberation (Groups S1, S2, S3, and S4, resp.; *n* = 4 per subgroup). In the third part of the experiment, eighty-eight animals were randomly allocated to one of the four groups: the sham group (Group S), the OALT group in which rats were administrated saline as a control (Group NS), the OALT group in which rats were administrated siRNA expression vector also as a control (group NC), and the OALT group in which rats were administrated TLR4 siRNA (group siRNA). The rats (*n* = 14 per group) were used to assess survival rates. Then the remaining rats (*n* = 8 per group) were killed and samples were collected at 8 hrs. All rats were anesthetized with pentobarbital (30 mg/kg body weight, i.p.). Tracheal intubation was performed with inserting a 14-gauge intravenous catheter which was then connected to small animal ventilators (ALC-V9A; Shanghai Alcott Biotech Co., Ltd., Shanghai, China). Rats were mechanically ventilated with a standard tidal volume (10 mL/kg) ventilation protocol (inspiratory/expiratory ratio of 1 : 1; respiratory rate 50 breaths/min; 100% oxygen). All the animals were killed with deep anesthesia and lung tissues and blood samples were collected for dynamic analysis of pulmonary function, histopathology, TLR4 gene and protein expression, and cytokines.

### 2.7. Arterial Blood Gas Analysis

The partial pressure of oxygen (PaO_2_) was measured using an iSTAT portable blood gas analyzer (G7+ chips, Abbott Laboratories, USA) according to the provided manufacturer's protocol.

### 2.8. Water Content of Lung in Rats

For assessing the severity of alveolar edema, the wet weight of the superior lobe of the right lung was accurately measured and baked to constant weight (80°C, 24 hrs) to calculate the water content of lung as follows: water content = (lung wet weight − lung dry weight)/lung wet weight × 100 [19].

### 2.9. Lung Histologic Evaluation in Rats

For histologic evaluation, fresh lung sections were fixed in 10% buffered formalin and embedded in paraffin; sections (4 *μ*m) were stained with hematoxylin and eosin. A pathologist who was blinded to research group assignment analyzed the samples and determined the levels of lung injury according to a scoring system as described by Derks and Jacobovitz Derks [20].

### 2.10. Reverse Transcription and Quantitative Real-Time PCR

Total RNA was extracted from lung samples by TRIzol (Invitrogen) according to the provided manufacturer's protocol. Reverse transcription (RT) of the lung samples was performed according to the introduction of TaqMan MicroRNA Reverse Transcriptase Kit (Life Technologies, Gaithersburg, MD, USA). Quantitative real-time PCR (qRT-PCR) was performed using the TaqMan Universal PCR Master Mix, NoAmpErase UNG (Life Technologies), and the Light Cycler 480 system (Roche, Switzerland). The relative expression of the target genes (TLR4 and *β*-actin) was calculated by the 2^−ΔΔCt^ method. The qPCR primers were as follows:

 TLR4 forward primer: 5′-CCTAGGCACCAGGGTGTGAT-3′.
 TLR4 reverse primer: 5′-TTGGTGACAATGCCGTGTTC-3′.
 
*β*-actin forward primer: 5′-TGCTTGTGGTAGCCACTGTA-3.′
 
*β*-actin reverse primer: 5′-CCTCATTCTGGCTCGAGTAG-3′.

### 2.11. Western Blot Analysis

Expression of TLR4 was assayed using western blotting according to our previous study [17]. The primary antibodies used were monoclonal rabbit anti-mouse TLR4 (1 : 500 dilution, Santa Cruz Biotechnology, USA). The secondary antibody used was goat anti-rabbit HRP-conjugated IgG (1 : 2000 dilution, Cell Signaling Technology, USA). Protein band density was measured using UVP Labworks Imaging Analysis software (UVP Labworks, Upland, CA, USA). The density measurement was correlated to protein expression and normalized to *β*-actin.

### 2.12. Immunofluorescent Assay

Frozen sections of lung tissue were immunofluorescent stained using polyclonal rabbit anti-mouse NF-*κ*B P65 (1 : 1000 dilution, Cell Signaling Technology, USA) according to our previous study [21].

### 2.13. Assay for Human Plasma TLR4 in Polymorphonuclear Leukocytes by Flow Cytometry

Flow cytometry was performed using phycoerythrin-Cy5-labelled monoclonal antibody for TLR4 (eBioscience, San Diego, USA) of human according to the provided manufacturer's protocol [22].

### 2.14. Assay for Human Serum TNF-*α*, IL-1*β*, and Rat Lung Tissue and Serum TNF-*α* and IL-1*β* by Enzyme-Linked Immunosorbent Assay (ELISA)

The contents of TNF-*α* and IL-1*β* in human serum were measured by ELISA kits (RapidBio Lab, California, USA) according to the provided manufacturer's protocol of the kits. The levels of TNF-*α* and IL-1*β* were expressed as pg/mL. Lung tissues harvested from rats were homogenized with cold normal saline and then centrifuged at 4000 r/min for 15 minutes. Supernatants were transferred into fresh tubes for detection. The contents of TNF-*α* and IL-1*β* in lung tissue and serum were measured by respective ELISA kits (R&D Systems, Minneapolis, MN, USA). The levels of TNF-*α* and IL-1*β* were expressed as pg/g protein in lung tissue and pg/mL in serum sample.

### 2.15. Detection of Myeloperoxidase (MPO) Activity

MPO activity in lungs was used as an indicator of peripheral blood polymorphonuclear leukocytes (PMNs) infiltration. Using a method previously described [23], MPO activity was detected and defined as the quantity of enzyme required degrading 1 mmol of H_2_O_2_ at 37°C and was expressed in mU/100 mg wet tissue.

### 2.16. Survival and Prognostic Analysis

To observe whether or not TLR4 gene silencing improves the prognosis of liver transplantation, we observed survival rates. The rats (*n* = 14 per group) receiving the same protocols were used to assess survival rates. From the onset of reperfusion, animals were monitored via video recording for 24 hrs. Two hours after reperfusion, the survivors were transferred to their individual cages and allowed free access to food and water.

### 2.17. Statistical Analysis

All data were analyzed using* SPSS 12.0* software (SPSS, Chicago, Illinois). The normal distribution data were expressed as mean ± standard deviation or SEM. The differences among the groups were evaluated by using one-way analysis of variance (*ANOVA*) followed by the* Tukey* test. Correlation between different variables was assessed by Spearman's coefficient. Differences were determined to be statistically significant at *P* < 0.05.

## 3. Result

### 3.1. The Levels of TLR4 on PMNs and Serum Inflammatory Cytokines Were Elevated in Patients with ALI following OLT

PMNs migration in circulation system has been shown to be involved in lung inflammatory injury induced by LPS. TLR4 takes part in the initiation of inflammation. We observed TLR4 expression on PMNs from peripheral blood of patients received OLT in order to find out the relation between TLR4 and ALI induced by OLT. We first confirmed the different levels of TLR4 on PMNs and the associated inflammatory cytokines in patients (basic information in Table 1) who received OLT surgery with or without ALI. As shown in Table 1, patients with ALI exhibited more ascites (*P* = 0.01) and longer anhepatic phase (*P* < 0.032). Moreover, severe fluctuating of circulatory volume is assumed as more blood loss volume (*P* = 0.034) and total output (*P* = 0.003) which lead to more infusion of red blood cell (*P* = 0.021) existing in patients with ALI compared to the non-ALI ones. Expression of TLR4 on PMNs was increased gradually following OLT, which was significantly higher in ALI group than that in non-ALI group (*P* < 0.05) at 24 hrs after neohepatic phase. Moreover, the TLR4 downstream cytokines TNF-*α* and IL-1*β* were synchronously increased after OLT, and ALI group exhibited significantly increased TNF-*α* and IL-1*β* level (*P* < 0.05) at 24 hrs after neohepatic phase (Table 2). These results indicated that inflammatory response especially TLR4 associated pathway was activated in patients with ALI following OLT.

### 3.2. ALI Induced by OALT in a Rat Model

**Figure 1:**
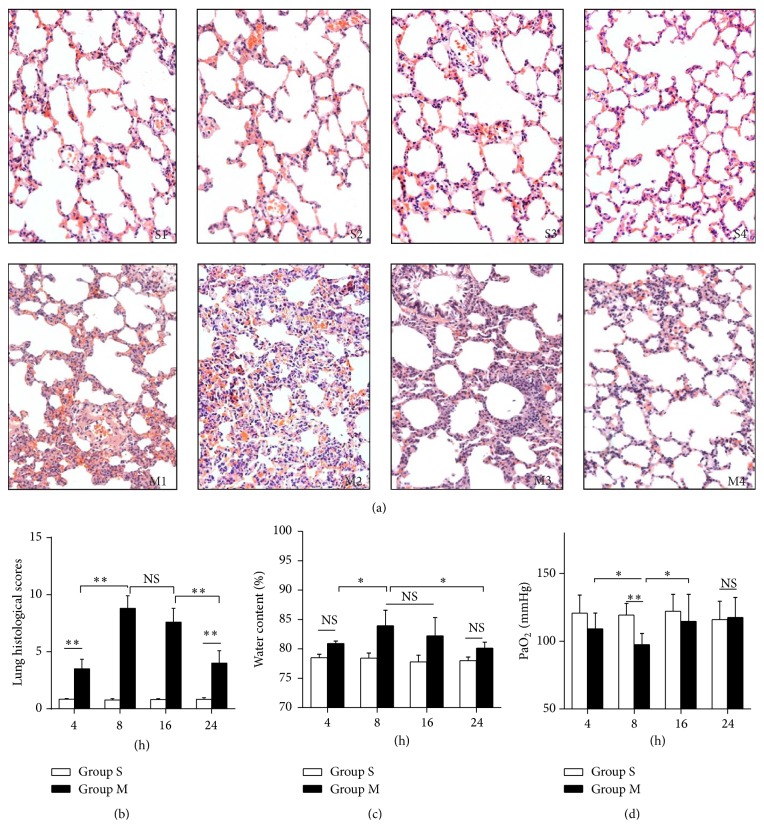
ALI induced by OALT in rat model. (a) Lung histology morphology. Lung sections were stained with hematoxylin and eosin (H&E) and visualized at 200x magnification. (b) Evaluation scores for lung histopathology analysis. (c) Water content of lung. (d) The arterial blood partial pressure of oxygen (PaO_2_). Groups M1, M2, M3, and M4 represented as collecting samples at liver reperfusion after 4 hrs, 8 hrs, 16 hrs, and 24 hrs, respectively. Values were expressed as mean ± SEM. *n* = 4 per group. NS: no statistical significance; ^*∗*^ *P* < 0.05, ^*∗∗*^ *P* < 0.01,* one-way ANOVA* with* Tukey* test.

In order to investigate the underlying mechanism of ALI following OLT, the OALT model was involved in our present study. The model could well mimic the hemodynamic change during liver transplantation (Table 3). Furthermore, we detected pathological and functional change in the injured lung at 4 hrs, 8 hrs, 16 hrs, and 24 hrs following OALT.  Table 4 shows no statistical significance in body weight and anhepatic phase duration time of the animals. As a result, we found collapse of pulmonary architecture; severe infiltration of polymorphonuclear and mononuclear cells into intra-alveolar, interstitial, and edematous interstitial space existed (Figure 1(a)) in rats that received OALT, accompanied with severe lung edema and dysfunctional pulmonary blood gas barrier which reflected in higher water content of lung (*P* < 0.05, Figure 1(c)) and lower PaO_2_ (*P* < 0.05, Figure 1(d)), which were correlated with increased pathological injury score (*P* < 0.05, Figure 1(b)). And these changes peaked at 8 hrs after reperfusion while they gradually recovered after 24 hrs. These above results showed that ALI occurred rapidly after OALT and maximized at 8 hrs after liver transplantation.

### 3.3. Lung TLR4 Expression and Levels of TNF-*α* and IL-1*β* Were Increased following OALT

**Figure 2:**
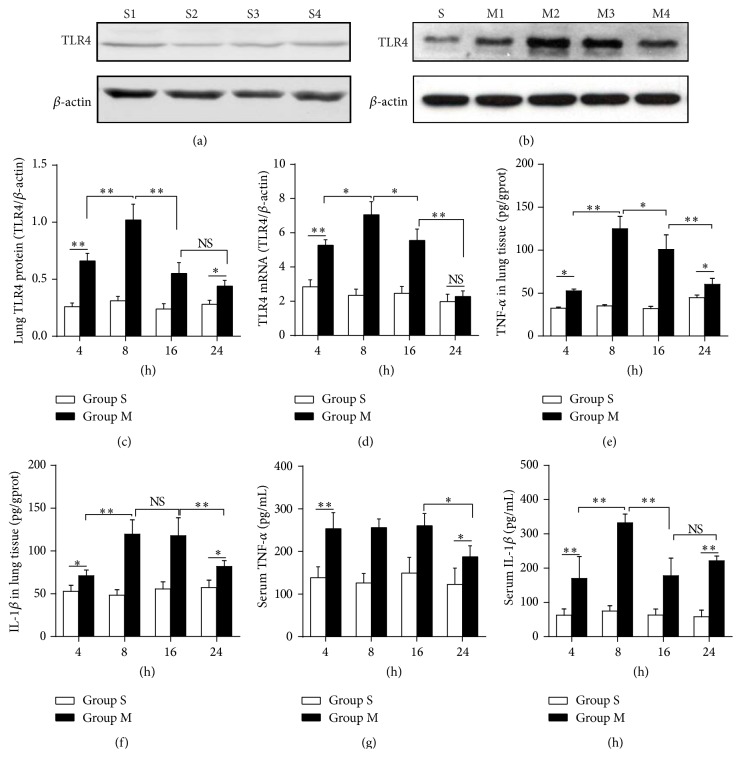
TLR4 gene and protein expression, concentration of TNF-*α* and IL-1*β*, and MPO activity in lung tissue or serum during OALT in a rat model. (a–c) TLR4 protein expression in lung tissue. (d) TLR4 mRNA expression in lung tissue. (e-f) TNF-*α*, IL-1*β* concentrations in lung tissue. (g-h) TNF-*α*, IL-1*β* concentrations in serum. Groups M1, M2, M3, and M4 represented as collecting samples at liver reperfusion after 4 hrs, 8 hrs, 16 hrs, and 24 hrs, respectively. Values are expressed as mean ± SEM. NS: no statistical significance; ^*∗*^ *P* < 0.05, ^*∗∗*^ *P* < 0.01,* one-way ANOVA* with* Tukey* test.

To investigate the underlying mechanism that accounted for ALI induced by OALT, we detected the TLR4 and its downstream cytokines which play important roles in mediating inflammatory cascades. As shown in Figures 2(a)–2(c), the mRNA and protein level of TLR4 in lung tissue were simultaneously elevated from 4 hrs after reperfusion and peaked at the timepoint of 8 hrs while they gradually decreased from 16 hrs after reperfusion. Furthermore, OALT obviously increased the level of TNF-*α* and IL-1*β* in lung tissue or serum from OALT groups, as illustrated in Figures 2(d)–2(h). Interestingly, we also found that the level of TNF-*α* and IL-1*β* in lung tissue reached maximum at 8 hrs. By contrast, the increase of serum cytokines seemed to be earlier evident as TNF-*α* reached maximum at 4 hrs while concentration of IL-1*β* peaked at 8 hrs. These results indicated that TLR4 and its associated downstream cytokines might act as the mediator of ALI progress which was obviously injured at 8 hrs after reperfusion, and this representative timepoint was chosen for further studies.

### 3.4. Knockdown of TLR4 Reduced NF-*κ*B p65 Nuclear Translocation in Injured Lung following OALT

**Figure 3:**
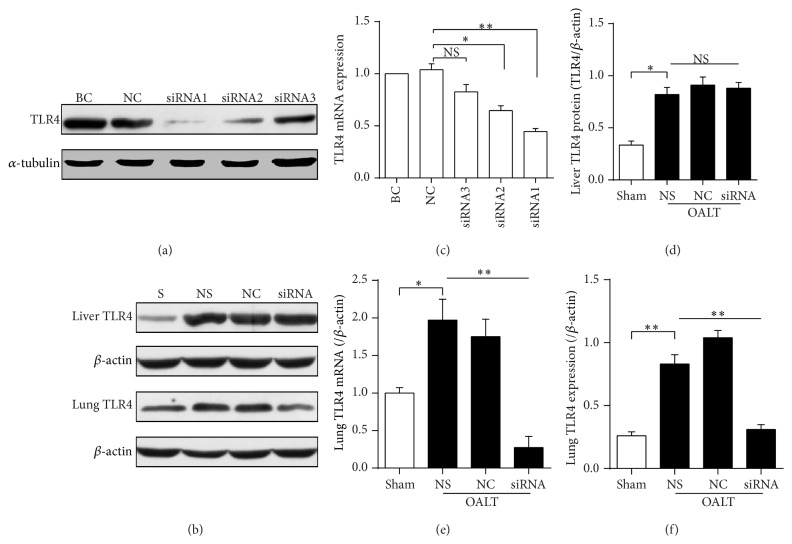
The effect of TLR4 siRNA on OALT-induced ALI in rats. (a) and (c) TLR4 gene and protein expression during screening for siRNAs. Groups BC, NC, siRNA1, siRNA2, and siRNA3 in A were treated with vehicle, negative control siRNA, siRNA1, siRNA2, and siRNA3, respectively. (b) and (d) showed the effects of TLR4 siRNA on TLR4 protein expression in liver tissue. (b), (e), and (f) showed the effects of TLR4 siRNA on TLR4 mRNA and protein expression in lung tissue. Groups S, NS, NC, and siRNA in (b) to (d) were treated with nothing, saline, negative control siRNA, and targeting siRNA, respectively. Values were expressed as mean ± SEM. NS: no statistical significance; ^*∗*^ *P* < 0.05, ^*∗∗*^ *P* < 0.01,* one-way ANOVA* with* Tukey* test.

**Figure 4:**
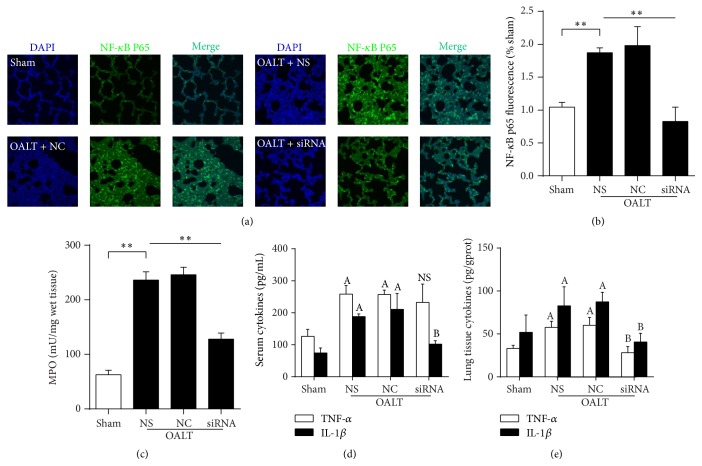
TLR4/NF-*κ*B P65 pathway was activated in injured lung following OALT. (a) and (b) showed immunofluorescent assay of NF-*κ*B P65 in lung tissue. (c) showed MPO activity in lung tissue. (d) and (e) showed TNF-*α* and IL-1*β* concentration in serum and lung tissue, respectively. Groups S, NS, NC, and siRNA were treated with nothing, saline, negative control siRNA, and targeting siRNA, respectively. Values were expressed as mean ± SEM. ^*∗*^ *P* < 0.0, ^*∗∗*^ *P* < 0.01, ^A^ *P* < 0.05 versus Group S, ^B^ *P* < 0.05 versus Group NC, NS means no significant difference versus Group NC,* one-way ANOVA* with* Tukey* test.

SiRNA administration, as a gradually mature technology, was used in our current study to knock down TLR4 induction. First, in HSC cell line, we tested three TLR4 siRNA sequences and determined that sequence 1 was the most effective in inhibiting TLR4 protein and mRNA expression (Figures 3(a) and 3(c)). Then, we confirmed effective knockdown of OALT-induced TLR4 protein expression in rat lung after intranasal siRNA administration. TLR4 siRNA significantly inhibited OALT-induced TLR4 protein (Figures 3(b) and 3(f)) and mRNA (Figure 3(e)) expression in rat lung lysates whereas nonspecific siRNA administration had no effects (Figure 3(b)) and intranasal siRNA administration had no effects on liver TLR4 expression (Figures 3(b) and 3(d)). Next, NF-*κ*B p65, as a downstream transcription factor of TLR4 pathway, was involved in verifying the effects of TLR4 siRNA on signaling transduction. As shown in Figures 4(a) and 4(b), NF-*κ*B p65 nuclear translocation was significantly increased in NC group (*P* < 0.05 versus sham) while it significantly decreased with TLR4 siRNA treatment (*P* < 0.05 versus group NC); by contrast, the expression of NF-*κ*B p65 in cytoplasm was opposite. Levels of lung and serum TNF-*α* and IL-1*β*, important makers of inflammation during OALT and OALT-induced ALI, were also decreased in rats administrated TLR4 siRNA compared with nonspecific siRNA (Figures 4(d)-4(e)). Results demonstrated that TLR4/NF-*κ*B pathway which mediated lung inflammation was activated by OALT.

### 3.5. Knockdown of TLR4 Attenuated MPO Activity

PMNs migration plays an important role in the progress of ALI. MPO is an indication of increased neutrophil infiltration. Rats administrated TLR4 siRNA exhibited lower concentration of myeloperoxidase in injured lung tissue than rats administrated nonspecific siRNA after OALT (Figure 4(c)). This result demonstrated that PMNs migrated to injured lung following OALT.

### 3.6. Knockdown of TLR4 Attenuated ALI Induced by OALT

**Figure 5:**
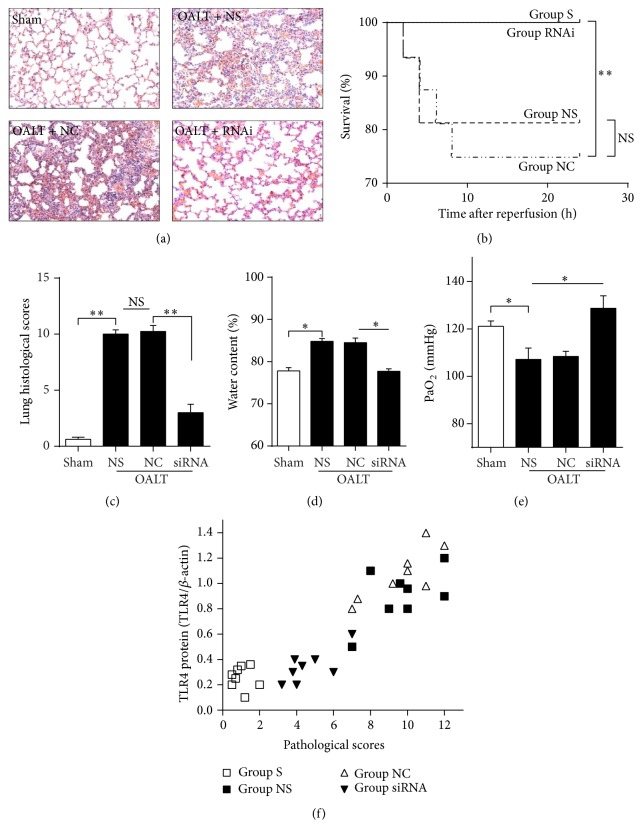
TLR4 siRNA reduced lung injury and resulted in an improvement of pulmonary function and rats survival. The effect of TLR4 siRNA on lung pathological change (a and c). Effect of TLR4 siRNA on survival (b). Survival time is calculated from the beginning of liver reperfusion. Results were compared by Kaplan-Meier log rank test and Fisher exact test, *n* = 14. (d) and (e) showed the effect of TLR4 siRNA on pulmonary function evidenced by lung water content and PaO_2_. (f) showed correlations between TLR4 protein levels and pathological scores. Groups S, NS, NC, and siRNA were treated with nothing, saline, negative control siRNA, and targeting siRNA, respectively. Values were expressed as mean ± SEM. NS: no statistical significance; ^*∗*^ *P* < 0.05, ^*∗∗*^ *P* < 0.01,* one-way ANOVA* with* Tukey* test.

To determine the functional role of TLR4 expression on ALI induced by OALT, we administrated TLR4 siRNA before rats were subjected to OALT. As shown in Figure 5(b), we found that knockdown of TLR4 significantly decreased mortality compared with rats given nonspecific siRNA in the OALT model (*P* < 0.05). These differences in mortality correlated with decreased lung injury parameters after OALT, evident by significantly decreased pathological scores (Figures 5(a) and 5(c)) and water content of lung (Figure 5(d)) with increased arterial PaO_2_ (Figure 5(e)) compared with group NC (*P* < 0.01). Furthermore, TLR4 protein levels were positively correlated with lung pathological scores (*r* = 0.8523; *P* < 0.001), which indicated the important role of TLR4 activation in the pathophysiologic process of ALI induced by OALT. These results indicated that TLR4 induction served as an important response to early stage of ALI induced by OALT* in vivo*.

## 4. Discussion

Clinical outcome in the present study showed that high level of TLR4 expression in circulating PMNs existed in patients with ALI after OLT, accompanied with high level of cytokines, providing clues of TLR4 overexpression involved in ALI induced by OLT. Lung inflammatory injury was induced by OALT in a rat model, evidenced by the increase of pathological scores and the release of cytokines along with decreases in PaO_2_ and the high mRNA and protein expression of lung tissue TLR4. These changes were more obvious at 8 hrs after OALT. Silencing TLR4 expression reduced the nuclear transfer of subunit P65 and attenuated ALI, after OALT. These clinical and experiment results indicate that TLR4 may contribute to ALI induced by liver transplantation and that TLR4 might be a potential intervention target.

Liver transplantation causes severe trauma and complex pathophysiological alterations, such as hemodynamic instability, liver and intestinal ischemia-reperfusion (I/R) injury, and the subsequent bacterial or endotoxin translocation and neutrophil infiltration due to I/R injury, leading to large amount of cytokines production and multiple inflammatory pathway activation [24, 25]. Secondly, these mixed injury factors result in the high incidence of postoperative complications. Lung is considered an early and vulnerable remote organ affected by OLT, and ALI occurs after OLT in a high incidence rate [3]. ALI has been characterized as an acute inflammation with sequestration of neutrophils and the subsequent enzymatic products in lung, resulting in increased microvascular permeability and then interstitial and pulmonary edema [26]. In our study, proinflammatory cytokines TNF-*α* and IL-1*β* significantly increased in the circulation and lungs in rats with ALI after liver transplantation. The degree of lung injury was proportional to the TNF-*α* and IL-1*β* levels in rat lung tissue. These results suggested that proinflammatory cytokines flooding, which may be due to neutrophil infiltration and multiple inflammatory pathway activation, played critical roles in amplifying lung inflammation and injury.

As an important member of the TLR family, TLR4 is expressed on multiple cell types [27]. TLR4 is especially enriched in monocytes-macrophages, neutrophils, and endothelial cells that are closely related to the inflammatory response [28]. TLR4 overexpression has been shown in injured lung induced by trauma or LPS [29–31]. In the current study, we found higher expression of TLR4 on circulating PMNs and higher levels of cytokines existed in patients with ALI than those in non-ALI patients. Interestingly, level of TLR4 expression on circulating PMNs was increased persistently even at 24 hours after neohepatic phase in ALI patients, indicating that TLR4 expression on circulating PMNs was closely related to prognosis of patients at the early stage of reperfusion. Also, the TLR4 level was raised transiently in non-ALI patients, which may be due to an acute phase response. On the other hand, the migration and activation of neutrophil have been demonstrated to initiate and aggravate the lung inflammatory response induced by traumatic factors or endotoxin [32, 33]. Neutrophils infiltration can be reflected by MPO activity which was released by the intravascular degranulation of neutrophils [34]. We also found high level of MPO activity in injured lung tissue following OALT in the animal experiments, indicating that neutrophils infiltration might play a critical role in ALI induced by liver transplantation. Furthermore, TLR4 mRNA and protein expression were found to be dynamically upregulated in lung tissues, which coincided with significant increases in cytokines and enhanced MPO activity in rats. All these data are consistent with the interpretation that neutrophils infiltration and upregulation of TLR4 expression participate in the development of ALI during OLT. Furthermore, recently, TLR4 in endothelial cell was showed to be a key determinant of LPS-induced PMNs infiltration in lungs [35, 36]. TLR4 stimulation in endothelial cells induced rapid expression of cell surface adhesion proteins, resulting in increased neutrophils rolling and firm adhesion [36]. Massive numbers of PMNs accumulated in lungs in response to activation of TLR4 [37]. However, study showed impaired PMNs recruitment into the alveolar space in TLR4 null mice [38]. Moreover, PMNs TLR4 has also been reported to regulate interaction of PMNs with activation of endothelial cells [39]. Another study using chimeric mice proved that endothelial cell TLR4 rather than PMN TLR4 plays the dominant role in LPS-induced PMNs infiltration in lungs [36]. However, our study differs in that we examined the role of TLR4 in a model of lung injury induced by multiple factors from liver transplantation which was more in line with clinical practice and both endothelial cell TLR4 and PMN TLR4 might be involved in the progress of ALI induced by OALT.

Studies have confirmed that TLR4 could activate transcription factors nuclear factor *κ*B (NF-*κ*B), amplify inflammatory responses, and lead to the injury (including lung injury) of multiple tissues and end-organs induced by endotoxin, massive transfusion, and trauma [40, 41]. The TLR4/NF-*κ*B pathway activation has an effect on the production of inflammatory cytokines and chemokines, resulting in PMNs infiltration and the subsequent exaggerated inflammatory response [42]. Excess production of proinflammatory cytokines such as TNF-*α* and IL-*β* can deteriorate the inflammatory process. In our current study, TNF-*α* and IL-*β* acted as important response of inflammation. Although there are various pathways involved in cytokines synthesis, TLR4/NF-*κ*B pathway has been proven to be playing an important role in cytokines (TNF-*α* and IL-*β*) productions; our current study also demonstrated that the level of cytokines could reflect effects on treatment of TLR4 siRNA, indicating the close relation between TLR4/NF-*κ*B pathway activation and downstream cytokines (TNF-*α* and IL-*β*) production. Interestingly, a recent study reported that TLR4 was a receptor for HMGB1-mediated ALI after liver partial ischemia/reperfusion injury [8]. This finding also supported our experimental hypothesis.

Various studies have shown that the high expression of TLR4 on separated lung cells plays a role in ALI [43–46]. Although we found a high expression of TLR4 in ALI, we did not investigate the cell type positioning of TLR4 siRNA, which will be involved in our study in the future. In addition, study has shown that activation of TLR4 alone or in conjunction with TLR2 could attenuate lung injury, associated with promotion of prosurvival effects on alveolar epithelium cell [47]. Similarly, TLR3-deficient mice were also showed to be protected from lung damage after hyperoxic exposure* in vivo*, accompanied with increased survival rate [48]. Collectively, the above finding implies that the role of TLRs in ALI is highly contextual. And, in the present study, our data support the idea that TLR4 is the major pathway that mediates the OALT-induced ALI. However, the role of TLR2 and TLR3 in the OALT-induced ALI needs to be explored in future studies to make the mechanism of TLRs acting on ALI more clear.

In conclusion, our study provides compelling evidence that the upregulation of TLR4 was involved in the ALI that occurred in rats after liver transplantation and downregulation of TLR4 by siRNA was proven to be an important therapy for ALI induced by liver transplantation. Thus, the potential manipulation of TLR4 activity to control lung injury may permit advances in liver transplantation. Furthermore, various TLR4 antagonists are under development for indications like septic shock [49], suggesting that such strategies may effectively reduce ALI early in the course of the disease and TLR4 may also act as an important target in the development of clinical drug for ALI treatment.

## Figures and Tables

**Table 1:** Clinical characteristics of the ALI group and non-ALI group during the peri-OLT period in patients.

Clinical characteristics	ALI group	Non-ALI group	*P* values
*N*	8	14	—
Age	47 ± 8	45 ± 12	0.801
Weight	63 ± 9	62 ± 6	0.801
Sex (M : F)	7 : 1	12 : 2	0.999
CTP scores (A : B : C)	4 : 2 : 2	6 : 2 : 6	0.727
Concentrated red blood cells (mL)	1313 ± 482	786 ± 467	0.021^*∗*^
Fresh frozen plasma (mL)	2200 ± 307	2050 ± 620	0.532
Platelets (mL)	125 ± 71	114 ± 66	0.725
Cryoprecipitate (mL)	969 ± 281	855 ± 305	0.399
Albumin (mL)	303 ± 156	318 ± 101	0.781
Total input (mL)	7978 ± 999	7639 ± 1679	0.611
Blood loss volume (mL)	3925 ± 1136	2700 ± 1253	0.034^*∗*^
Urine output (mL)	1543 ± 580	1630 ± 809	0.793
Ascites (mL)	1150 ± 500	100 ± 50	0.01^*∗∗*^
Total output (mL)	7493 ± 2494	4537 ± 1571	0.003^*∗∗*^
Total operation time (min)	406 ± 61	390 ± 70	0.601
Preanhepatic phase (min)	91 ± 28	94 ± 27	0.809
Anhepatic phase (min)	59 ± 37	36 ± 5	0.032^*∗*^
Neohepatic phase (min)	259 ± 33	259 ± 57	0.978

Compared with the non-ALI group, ^*∗*^ *P* < 0.05, ^*∗∗*^ *P* < 0.01.

**Table 2:** Perioperative differences in the expression of TLR4 on PMNs and the serum concentrations of TNF-*α* and IL-1*β* between patient groups.

Groups		T_1_	T_2_	T_3_	T_4_
ALI (*n* = 8)	TLR4 (MIF)	23.87 ± 13.33	36.59 ± 24.52	43.59 ± 31.05	75.54 ± 34.05^bc^
	TNF-*α* (pg/mL)	73.53 ± 25.72	94.13 ± 40.97	94.80 ± 44.17^a^	289.43 ± 83.04^ac^
	IL-1*β* (pg/mL)	57.52 ± 17.85	62.44 ± 24.09	96.58 ± 41.28	197.01 ± 58.85^ac^

Non-ALI (*n* = 14)	TLR4 (MIF)	27.25 ± 11.63	33.05 ± 13.96	38.08 ± 11.42	22.25 ± 7.97
	TNF-*α* (pg/mL)	129.91 ± 80.55	139.67 ± 83.32	117.79 ± 49.52	163.67 ± 76.35
	IL-1*β* (pg/mL)	51.94 ± 21.78	44.62 ± 23.40	29.97 ± 22.77	67.63 ± 26.15

MIF: mean fluorescence intensity; T_1_: after induction of anesthesia; T_2_: 1 hour after anhepatic phase; T_3_: 4 hours after neohepatic phase; and T_4_: 24 hours after neohepatic phase. Data were expressed as the mean ± SD or median (interquartile range); ^a^ *P* < 0.05, ^b^ *P* < 0.01 versus T_1_; ^c^ *P* < 0.05 versus non-ALI.

**Table 3:** Hemodynamic changes during OALT in rats.

		Baseline	Vascular clamped	Vascular unclamped	5 min after vascular unclamped
Heart rate (BPM)	Group sham	362 ± 7	—	—	—
	Group model	361 ± 3	446 ± 9^a^	410 ± 11^bd^	225 ± 26^ace^

MAP (mmHg)	Group sham	92 ± 3	—	—	—
	Group model	93 ± 3	47 ± 5^a^	61 ± 5^bc^	42 ± 3^af^

BPM: beat per minute. Data are expressed as mean ± SD, *n* = 3. ^a^ *P* < 0.01, ^b^ *P* < 0.05, versus baseline; ^c^ *P* < 0.01, ^d^ *P* < 0.05, versus vascular clamped; ^e^ *P* < 0.01, ^f^ *P* < 0.05, versus vascular unclamped.

**Table 4:** Rat body weight and anhepatic phase duration time in studied groups.

Group	Weight (g)	Anhepatic phase (min)
S1	237.3 ± 21.0	—
S2	233.6 ± 15.6	—
S3	241.3 ± 14.1	—
S4	242.2 ± 15.2	—
IR4 h	240.6 ± 13.7	19.9 ± 0.6
IR8 h	229.1 ± 22.0	20.0 ± 0.5
IR16 h	243.1 ± 17.5	20.1 ± 0.6
IR24 h	243.1 ± 13.3	20.1 ± 0.6

Data are expressed as mean ± SD, *n* = 8.
